# Integrating CRISPR technology into neurotrauma research: opportunities and challenges in addressing synaptic dysfunction after TBI

**DOI:** 10.1097/MS9.0000000000003998

**Published:** 2025-09-29

**Authors:** Hameed Ullah, Rania Ahmad, Maliha Khalid, Muhammad Talha, Aminath Waafira

**Affiliations:** a Department of Medicine, Quaid-E-Azam Medical College, Bhawalpur, Pakistan; b Department of Medicine, Jinnah Sindh Medical University,Karachi, Pakistan; c Department of Medicine, King Edward Medical University, Lahore, Pakistan; d School of Medicine, The Maldives National University, Malé, Maldives

**Keywords:** CRISPR/cas9, neurotrauma research, synaptopathies, traumatic brain injury

## Abstract

Traumatic brain injury (TBI) is a major global cause of neurological disability, with long-term consequences that include synaptic dysfunction and rare synaptopathies. The CRISPR/Cas9 gene-editing system has emerged as a powerful tool in neurotrauma research due to its efficiency in gene knockout, knock-in, transcriptional regulation, and base editing. Recent studies demonstrate its potential in modulating synaptic proteins such as PSD-95, Synaptophysin, GluN1, and GluA2, providing insight into mechanisms of TBI-related synaptopathies. While CRISPR offers remarkable opportunities to advance our understanding and treatment of neurotrauma, challenges remain, including off-target effects, error-prone repair, inefficient delivery systems, and immunogenicity. Overcoming these barriers through improved epigenetic tools, targeted non-viral delivery methods, and multiplexed modulation strategies may enable more precise interventions. Ethical oversight and long-term safety monitoring will be critical to harness CRISPR’s full therapeutic potential for addressing TBI-associated synaptic dysfunction.

*To the editor,*

Traumatic brain injury (TBI) is a leading cause of death and neurological disability worldwide, resulting from increased use of vehicles in low-income countries. Traumatic brain injury refers to normal brain dysfunction due to mechanical force to the head. It affects neurons and synapses and can lead to chronic and rare synaptopathies^[1]^. TBI pathophysiology encompasses mechanical insult (primary injury) and various secondary injuries, including excitotoxicity, mitochondrial dysfunction, oxidative stress, neuroinflammation, and apoptotic cell death^[2]^. In this LTE, we highlight the integration of CRISPR-based approaches into future neurotrauma research.

CRISPR is a 3rd-generation gene editing tool, a better version than all the previous techniques. This system is simple and efficient in multiple gene editing techniques, including gene knockout, gene knock-in, transcriptional regulation, and base editing. The CRISPR/Cas9 tool identifies the target gene by using single-guide RNA (sgRNA). These RNA guides the DNA double strands to be cleaved into double-strand breaks (DSB) by Cas9 nuclease activity. After DNA damage, spontaneous repair can occur, ultimately leading to gene editing. Application of CRISPR in neurological diseases such as Alzheimers, Parkinson’s, Huntington’s, glioblastoma, epilepsy, and amyotrophic lateral sclerosis has significantly revealed the underlying mechanisms, thus providing ways for preventing and treating synaptopathies^[3]^.

Recent studies have highlighted the role of CRISPR-based modulation of synaptic proteins whose disruptions can lead to synaptic dysfunctions and rare synaptopathies induced by TBI. A study demonstrates the role of CRISPR/Cas9 using the nucleofection technique in successfully knocking out a postsynaptic protein, PSD-95, and a presynaptic protein, Synaptophysin (Syp), in cultured neurons derived from rat cortex and hippocampus^[4]^. Another study explains the potency of CRISPR/Cas9 in hippocampal slice culture to inactivate two key synaptic proteins, GluN1 and GluA2. After removing them, these proteins stopped their function completely, showing the effectiveness of CRISPR^[5]^.

Although CRISPR is considered a relatively simple and easy-to-use tool for gene editing and plays a remarkable role in the modulation of synaptic proteins, it is not without important challenges. It has some critical limitations, such as off-target mutagenesis, error-prone repair, ineffective delivery of CRISPR machinery, and immunogenic problems. These challenges collectively highlight both the strengths and shortcomings of CRISPR, emphasizing the need for further refinement and innovation. Simultaneously, they also define its potential role in shaping future research directions, where overcoming these barriers could unlock more reliable and safer therapeutic applications^[6]^.

In conclusion, to fully realize CRISPR’s potential in treating TBI-associated rare synaptopathies, future studies should focus on the use of advanced epigenetic tools, improved non-viral delivery of vectors into specific neuronal targets, and multiplexed modulation of synaptic proteins to study complex disorders like ASD and Schizophrenia. Ethical planning and long-term monitoring are essential from the start to ensure safe application, paving the way for a new era of genome editing. This letter to editor is in compliance with the TITAN guideline^[7]^.

## Data Availability

No data were generated for this manuscript.

## Research registration unique identifying number (UIN)

Name of the registry: N/A; Unique Identifying number or registration ID: N/A; Hyperlink to your specific registration (must be publicly accessible and will be checked): N/A.

## Peer and provenance statement

This article was not commissioned and was externally peer-reviewed.
